# The complete mitochondrial genome of *Parasitus fimetorum* (Berlese, 1904) (Arachnida: Parasitiformes: Parasitidae)

**DOI:** 10.1080/23802359.2022.2081944

**Published:** 2022-06-14

**Authors:** Huijuan Yang, Ting Chen, Wenge Dong

**Affiliations:** Yunnan Provincial Key Laboratory for Zoonosis Control and Prevention, Institute of Pathogens and Vectors, Dali University, Dali, China

**Keywords:** Parasitidae, *Parasitus fimetorum*, complete mitochondrial genome, phylogenetic analysis

## Abstract

For the first time, the complete mitochondrial genome of *Parasitus fimetorum* was sequenced. The mitochondrial genome is 14,619 bp in length and includes 13 protein-coding genes, 22 transfer RNA genes, two ribosomal RNA genes, and a control region. The base composition is 35.6% for A, 34.8% for T, 18.2% for G, and 11.4% for C. A phylogenetic tree based on the maximum likelihood (ML) method indicated that *Parasitus fimetorum* was clustered with *Parasitus wangdunqingi* within the family Parasitidae.

*Parasitus fimetorum* (Berlese, 1904) (Arachnida: Parasitiformes: Parasitidae) is a free-living mite in the family Parasitidae that feeds on edible fungus pests (Juha 2006; Bardgett and Van der Putten 2014). The mitochondrial genome, with its maternal inheritance, relatively high mutation rates, and lack of recombination, has been extensively used as genetic markers in molecular phylogenetic studies (Dong et al. 2021). The scarcity of mitochondrial genome data in the family Parasitidae has hampered the research of evolution among Parasitidae species. In this study, the complete mitochondrial genome of *P. fimetorum* was sequenced for the first time. This will lay the foundation for further research on the identification and biological evolution of the family Parasitidae species.

*Parasitus fimetorum* was collected from the body surface of *Apodemus chevrieri* (Mammalia: Rodentia: Muridae: Apodemus) in Lijiang City, Yunnan Province, China (26°57′N, 100°15′E). Specimen and vouchers of *P. fimetorum* were deposited at the Institute of Pathogens and Vectors, Dali University, China (voucher number: 163, Wenge Dong, dongwenge2740@sina.com). The DNeasy Blood and Tissue Kit (QIAGEN) was used to extract genomic DNA. Sequenced the complete mitochondrial genome on the Illumina platform. The mitochondrial genome of *P. fimetorum* was annotated using the software Geneious 11.1.5 (Kearse et al. 2012). The nucleotide sequences of *P. fimetorum* have been deposited in GenBank under accession number OK572962.

**Statement**: Capture of *Apodemus chevrieri* (Mammalia: Rodentia: Muridae: Apodemus) was performed in accordance with the guidelines and regulations approved by the Animal Ethics Committee at Dali University (name: Dali University Ethics Committee; approval ID: MECDU-201806-11).

*Parasitus fimetorum* mitochondrial genome is 14,619 bp in length and includes 13 protein-coding genes (*nad1*-*nad6*, *nad4L*, *cox1*-*cox3*, *cob*, *atp6*, *atp8*), 22 transfer RNA genes, two ribosomal RNA genes, and a control region. The overall base composition is 35.6% for A, 34.8% for T, 18.2% for G, and 11.4% for C. 22 genes (nine protein-coding genes and 13 tRNA genes) were coded on the majority strand (J-strand). The other 15 genes were coded on the minority strand (N-strand). Except for *nad1* and *nad5*, which start with GTG codons, other protein-coding genes start with standard ATN codons (5 ATT, 4 ATG, and 2 ATA). Four protein-coding genes (*cox2*, *cox3*, *cob* and *nad5*) have an incomplete stop codon (T), and the other nine protein-coding genes have complete stop codons (TAA or TAG). With the exception of *trnS_1_* (anticodon GCU), other tRNAs in *P. fimetorum* can form the typical clover secondary structure.

Based on 13 protein-coding genes sequences, a phylogenetic tree between *P. fimetorum* and the other 13 species was constructed using MEGA X (Kumar et al. 2018) software with the maximum likelihood method (Figure 1). Clade support was assessed using a nonparametric bootstrap with 1000 replicates.The nucleotide substitution model was JTT + F + G. *Carcinoscorpius rotundicauda* and *Limulus polyphemus* were selected as outgroups in the phylogenetic tree. Phylogenetic analysis showed that species of the same family always preferentially cluster together. *Parasitus fimetorum* was clustered with *Parasitus wangdunqingi* within the family Parasitidae. *Parasitus fimetorum* mitochondrial genome will help us better understand the phylogenetic and evolution of the family Parasitidae mitochondrial genome.

**Figure 1.**
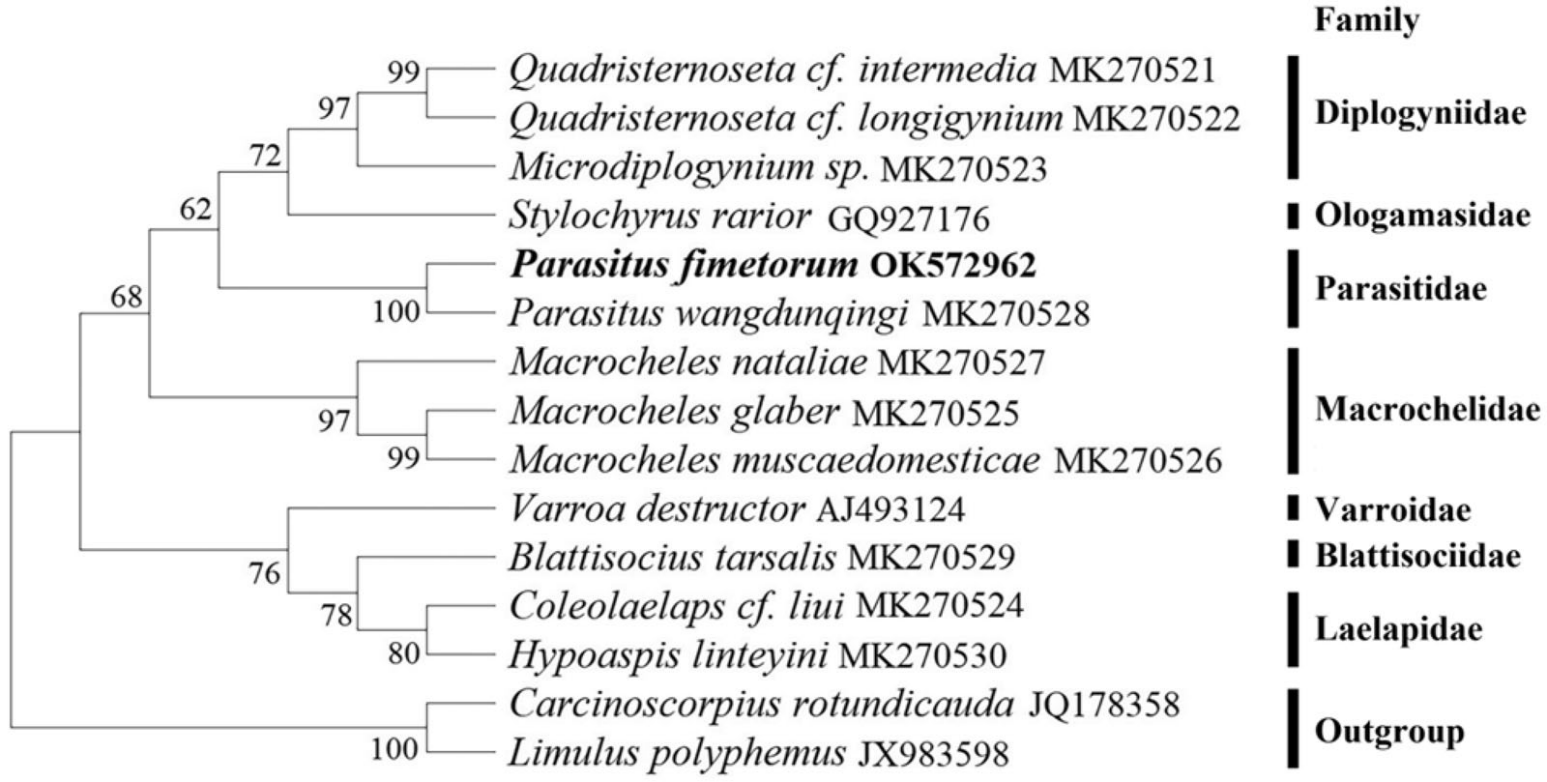
The phylogenetic tree was constructed using the maximum-likelihood (ML) based on 13 protein-coding gene sequences. Numbers on each branch indicate bootstrap values. Bolded and blackened vertical lines indicate species of the same family.

## Authors’ contributions

Huijuan Yang, Ting Chen and Wenge Dong designed the research. Huijuan Yang and Ting Chen performed the research. Material preparation, data collection were performed by Huijuan Yang and Ting Chen. Huijuan Yang and Wenge Dong analyzed the data. The first draft of the manuscript was written by Huijuan Yang. Wenge Dong critically revised the content of the paper. All authors have read and the final manuscript approval of the version to be published; and that all authors agree to be accountable for all aspects of the work.

## Data Availability

The genome sequence data that support the findings of this study are openly available in GenBank of NCBI at https://www.ncbi.nlm.nih.gov/, reference number OK572962. The associated BioProject, SRA, and Bio-Sample numbers are PRJNA808201, SRR18076603, and SAMN26036431 respectively.
